# Edge-Enhance YOLO for Steel Surface Defect Detection

**DOI:** 10.3390/jimaging12060259

**Published:** 2026-06-12

**Authors:** Renfei Li, Mingxiu Lin

**Affiliations:** College of Information Science and Engineering, Northeastern University, Shenyang 110819, China; lirf1@mails.neu.edu.cn

**Keywords:** surface defect detection, EDEN-YOLO, edge-enhance module, gated module

## Abstract

Surface defect detection is an important task for quality assurance in steel manufacturing. Although YOLO-style detectors are widely used due to their strong performance, they often struggle to accurately localize edge-dominant defects such as crazing and fine cracks. This limitation arises because such defects exhibit weak feature representations. In addition, their high-frequency structural details are progressively degraded during repeated downsampling. To address this issue, a YOLO-based detection framework named EDEN-YOLO is proposed. It incorporates an in-place Edge-Enhance module into the YOLOv8 baseline to improve structural sensitivity. Specifically, a Local Feature Enhancement (LFE) module is designed to capture edge-sensitive patterns. A Gated Module is further introduced to perform spatially selective recalibration of backbone features. This design enhances edge responses while suppressing noise. Experiments on the NEU-DET benchmark demonstrate the effectiveness of the proposed method. EDEN-YOLO achieves 80.5% mAP@0.5 on NEU-DET, showing an improvement over the reproduced YOLOv8 baseline while introducing a moderate increase in model complexity by 0.52M parameters and 1.3 GFLOPs. A supplementary evaluation on the GC10-DET dataset shows that EDEN-YOLO achieves 65.2% mAP@0.5, compared with 61.0% for the reproduced YOLOv8 baseline. The qualitative results show that the proposed module produces more compact feature responses.

## 1. Introduction

Surface defect detection is essential for ensuring product quality in the steel industry. Traditional quality control relies on manual inspection or traditional machine vision techniques. However, as summarized in comprehensive reviews [1], methods based on handcrafted features, such as edge and texture descriptors, often lack robustness under complex inspection conditions; for instance, different criteria may lead to different detection results, resulting in false negatives and false positives.

With the increasing adoption of deep learning in visual inspection, CNN-based detectors have become widely used for industrial defect detection. Early YOLO-based methods demonstrated the potential of one-stage detectors in balancing detection accuracy and inference efficiency for steel surface inspection [2]. However, standard YOLO-style detectors mainly rely on hierarchical semantic features extracted through repeated convolution and downsampling. For defects with clear regional appearance, such representations are usually sufficient. For edge-dominant or weak-contrast defects such as crazing and fine cracks, however, the structural details that define defect boundaries may become progressively weakened in deeper feature maps. This can lead to incomplete responses or inaccurate localization, especially when the defect contour is thin, fragmented, or mixed with background texture.

To mitigate such feature degradation while preserving high-accuracy performance, researchers have proposed various architectural enhancements, among which feature fusion designs are widely employed. LFF-YOLO introduces a feature fusion network to utilize multi-scale feature while reducing information loss during aggregation [3]. Besides steel surface detection, YOLO-AFK employs advanced fine-grained feature extraction to handle complex solder-joint defects, illustrating the effectiveness of refining backbone/neck representations for subtle industrial targets [4], thus providing a useful design paradigm to improve the perception of subtle targets. To leverage more modal information, Dual-backbone strategies have been explored to strengthen complementary feature learning. MBDNet-Attention-YOLO adopts a dual-backbone design with adaptive fusion and attention to improve steel surface defect detection [5]. While these methods can achieve high accuracy, they often rely on additive complexity such as dual backbones or heavy attention blocks, which not only increase architectural complexity, as evidenced by existing dual-branch feature extraction frameworks [6,7], but also lead to increased computational overhead and memory usage.

Another potentially useful cue for defect localization is edge-related structural information, which is often captured in low- and mid-level feature representations. An edge-aware multi-level interactive network was proposed to sharpen localization of defect edges in salient-object detection settings [8], resulting in clearer boundary delineation under complex conditions. Similarly, edge-guided multiscale fusion has been shown effective for small-target detection under cluttered backgrounds [9]. Related boundary-localization challenges have also been studied in remote sensing tasks. A scribble-based edge-aware learning method further shows that structural supervision can help improve contour fidelity with sparse annotations [10]. Moreover, progressive edge guidance has also been explored in unsupervised anomaly detection, which has been leveraged to synthesize more realistic industrial anomalies, emphasizing the general importance of edge priors in defect-related tasks [11]. These studies suggest that edge-related structural cues can complement semantic features when object or defect boundaries are difficult to distinguish. Such structural cues may serve as complementary information for defect detection, especially for weak-contrast or edge-dominant defects whose boundaries are difficult to localize using semantic features alone. Therefore, incorporating edge-aware information into YOLO-style detectors may be useful for improving the localization of subtle surface defects.

In this paper, **ED**ge **EN**hance **YOLO** (EDEN-YOLO) is proposed as a YOLOv8-based detection framework for steel surface defect detection. The proposed design integrates semantic depth with structural sensitivity via an in-place enhancement mechanism. Compared with dual-backbone designs [5] or edge-aware parallel-branch learning [8], we introduce a Local Feature Enhancement (LFE) module that extracts and emphasizes edge cues at the intermediate stage while preserving the original single-backbone topology. The main idea of EDEN-YOLO is to construct a compact edge-enhancement branch by integrating existing structural operations into the YOLO feature extraction process. To ensure robust optimization, we introduce a Gated Module unit that leverages edge-enhanced cues to recalibrate the backbone features via pixel-wise fusion, suppressing noise while highlighting defect contours.

Our primary contributions are summarized as follows:

- A compact in-place Edge-Enhance module is constructed by integrating edge-sensitive filtering, noise suppression, and gated feature recalibration into the YOLOv8 backbone, enabling structural enhancement without introducing an additional backbone or detection branch.
- A stage-aware Local Feature Enhancement (LFE) design is developed to strengthen edge-related structural cues and suppress redundant high-frequency responses through the coordinated use of Scharr-based edge extraction and Gaussian smoothing.
- A gated multiplicative fusion strategy is introduced to convert enhanced structural responses into a spatial gain map, allowing for selective recalibration of the original backbone feature instead of directly adding enhanced features to the main stream.
- Experiments on NEU-DET and supplementary evaluation on GC10-DET demonstrate that EDEN-YOLO improves the reproduced YOLOv8 baseline under controlled training protocols, while ablation studies validate the contribution of module placement, structural operators, gated fusion, and scaling strength.

The remainder of this paper is organized as follows: Section 2 reviews related work on industrial defect detection and edge-aware learning. Section 3 presents the proposed EDEN-YOLO architecture and its components. Section 4 describes the experimental setup and results on the NEU-DET benchmark and the supplementary GC10-DET evaluation. Finally, Section 5 concludes this paper and discusses future directions.

## 2. Related Work

### 2.1. YOLO-Based Methods for Industrial Defect Detection

The You Only Look Once (YOLO) framework has promoted the wide use of single-stage detectors in industrial applications. These models achieve a good trade-off between detection speed and accuracy [12]. In the domain of steel surface inspection, numerous YOLO-based variants have been proposed to address complex defect patterns and deployment constraints. Ref. [13] developed LMS-YOLO by introducing multi-scale mixed convolutions to achieve lightweight feature extraction, while their subsequent work, LDE-YOLO, incorporated a re-parameterized feature pyramid to enhance real-time performance [14]. Similarly, Ref.  [15] integrated polarized self-attention and a bidirectional feature pyramid network into YOLOv8 to improve strip steel defect detection. More recent studies include YOLO-SCM, which employs dynamic context modeling for real-time steel plate inspection [16], and FMV-YOLO, which utilizes adaptive fine-grained channel attention to enhance robustness in real-world industrial environments [17].

Beyond the YOLO family, alternative architectures have also demonstrated competitive performance. For instance, Ref.  [18] utilized adaptively spatial feature fusion in RetinaNet for steel defect detection. Transformer-based detectors have further advanced detection accuracy, with representative models including HCT-Det [19], Lightweight RT-DETR [20], MESC-DETR [21], and SH-DETR [22].

Despite these advances, these methods mainly focus on enhancing semantic representation or multi-scale aggregation, while fine-grained structural cues such as edge-dominant patterns are often weakened or overlooked. However, DETR-based models usually require longer convergence and more complex training strategies, which may limit their practicality in some industrial inspection scenarios.

### 2.2. Edge Perception and Structural Guidance

Defects such as cracks, inclusions, and crazing are typically characterized by irregular edges and weak contrast, making accurate localization challenging. Nevertheless, explicit structural cues (e.g., boundaries and edge responses) can provide complementary guidance for feature modeling. Early deep learning approaches, such as Holistically-Nested Edge Detection (HED), employed multi-layer supervision for pixel-level edge learning [23]. Building upon this idea, boundary/edge-aware networks [24,25] incorporated structural cues into semantic and salient object detection frameworks.

In remote sensing and complex visual environments, edge-guided mechanisms have been combined with multiscale modeling to refine object contours [26], while UEDG introduced uncertainty–edge dual guidance to improve robustness under ambiguous conditions [27]. Recently, a broader set of structural priors, including classical edge operators and multi-scale smoothing, has been integrated into deep models to enhance low-quality object perception [28]. Related studies also explored second-order/pyramidal structural representations (e.g., Laplacian-related formulations) for enhancing boundary fidelity and cross-modal alignment [29,30,31].

However, most edge-guided methods rely on parallel auxiliary branches or heavy structural modifications, which inevitably increase computational overhead and deployment complexity. Moreover, the integration of structural cues is often performed uniformly across feature levels, potentially amplifying irrelevant high-frequency responses and degrading robustness in complex industrial environments. Therefore, how to incorporate edge-sensitive structural information into detection frameworks in a selective manner remains an open problem.

### 2.3. Feature Fusion and Dual-Path Learning

Multi-scale feature fusion aims to reconcile high-level semantic information with low or middle level spatial details, which contains edge features, thereby improving the detection of scale-varying objects [32]. Existing studies have shown that combining multi-feature fusion strategies with attention mechanisms can effectively strengthen multi-scale representations [32].

In addition, dual-path learning has been explored for multi-source and multi-modal inputs. For example, the YOLOFuse framework integrates infrared and visible images to enhance detection robustness [33]. However, excessive enhancement in high-frequency components across all feature levels may amplify background noise and degrade generalization performance.

In industrial defect detection, where defects often exhibit subtle edge patterns under complex textures, designing a selective mechanism that balances semantic abstraction and structural detail remains a challenging task.

Therefore, existing methods either introduce heavy architectural modifications or fail to selectively enhance structural information. How to incorporate edge-sensitive features into detection frameworks in a stable and selective manner remains an open problem.

## 3. Method

In this section, a comprehensive explanation of the proposed EDEN-YOLO model is provided. Each key module in the network architecture is described, and their respective designs are clarified.

The enhancement is applied at an intermediate backbone stage where spatial resolution and semantic abstraction are well balanced. This allows structural cues to be reinforced while reducing the risk of amplifying irrelevant background textures. Unlike methods that introduce additional parallel backbones or independent detection branches [33], our design embeds the Edge-Enhance module into the existing YOLOv8-style feature flow. Therefore, EDEN-YOLO preserves the original topology and remains compatible with standard YOLO training and inference pipelines.

**Figure 1:**
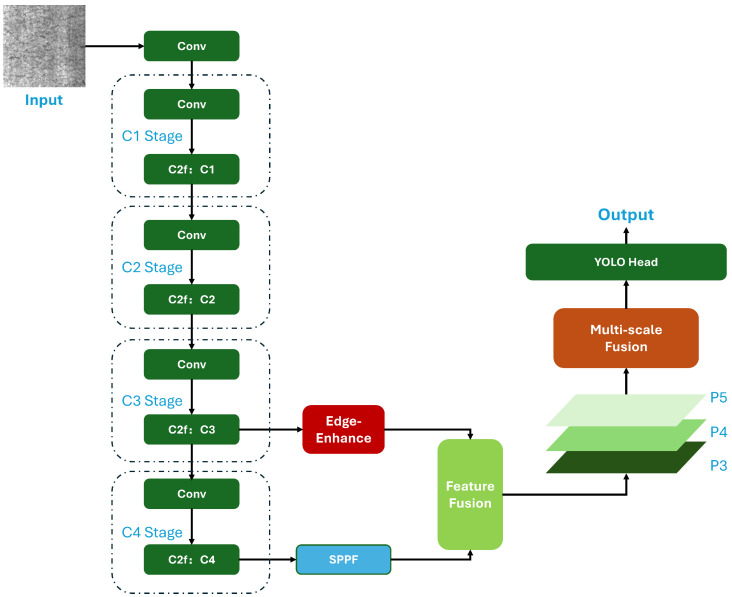
Overall architecture of EDEN-YOLO. The Edge-Enhance module is inserted after the C3 stage of the backbone to produce a recalibrated intermediate feature. The recalibrated feature is integrated into the YOLO neck together with the original hierarchical backbone features for multi-scale feature fusion, generating $\left\{P_{3},P_{4},P_{5}\right\}$ for detection.

The overall architecture of EDEN-YOLO is illustrated in Figure 1.

### 3.1. Overall Architecture

Given an input image *I*, EDEN-YOLO follows a YOLOv8-style architecture, as illustrated in Figure 1. The backbone first extracts hierarchical features through several convolutional and C2f stages. For clarity, these feature stages are denoted as C1, C2, C3, and C4, corresponding to progressively deeper representations. Among them, shallow stages preserve richer spatial details, while deeper stages contain stronger semantic abstraction.

The Edge-Enhance module is inserted at C3 Stage. This stage provides a favorable balance between spatial resolution and semantic representation, making it suitable for enhancing edge-dominant defect patterns without excessively amplifying shallow background noise or losing fine structural cues in overly deep features. Let $f_{0}$ denote the original C3 feature extracted by the backbone. The Edge-Enhance module predicts a spatial importance map *g* from the enhanced structural response and performs gated recalibration on the original feature:

(1) $$\tilde{f}=f_{0}⊙\left(1+\alpha g\right),$$

where ⊙ denotes element-wise multiplication, α is a scaling factor, and *g* denotes the spatial importance map predicted by the Edge-Enhance module. The detailed formulation of *g* is introduced in Section 3.2.2.

The recalibrated feature $\tilde{f}$ is then integrated into the YOLO neck for multi-scale feature fusion. The neck aggregates features at different resolutions and generates the fused detection features:

(2) $$\left\{P_{3},P_{4},P_{5}\right\}=F_{\mathrm{neck}}\left(\tilde{f},C\right),$$

where C denotes the original hierarchical backbone features used by the neck. Finally, the YOLO detection head predicts the detection results from the fused multi-scale features:

(3) $$\hat{Y}=D_{\mathrm{head}}\left(P_{3},P_{4},P_{5}\right).$$

### 3.2. Edge-Enhance Module

The proposed Edge-Enhance module is designed as a compact integration of existing structural operations and feature recalibration mechanisms. Its main purpose is to organize edge-sensitive enhancement, noise suppression, and gated recalibration into an in-place module suitable for YOLO-style steel defect detection. Specifically, the LFE branch extracts structural cues from the intermediate C3 feature using Scharr-based edge enhancement and Gaussian noise suppression, while the gated module converts the enhanced response into a spatial gain map for multiplicative recalibration of the original backbone feature. Unlike simple additive or residual fusion, the enhanced branch does not directly inject all responses into the main feature stream; instead, it selectively determines where the original backbone activations should be strengthened.

**Figure 2:**
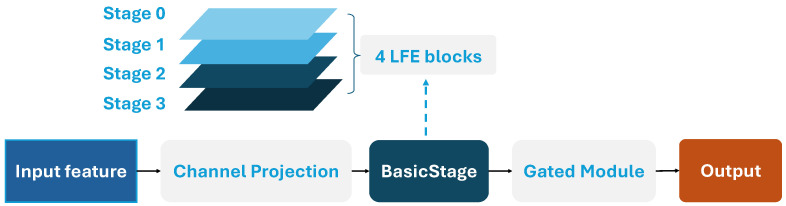
Structure of the proposed Edge-Enhance module. It consists of channel projection, a BasicStage containing multiple LFE blocks, and a gated module unit for in-place recalibration.

The detailed structure of the proposed Edge-Enhance module is shown in Figure 2. It follows a compact pipeline:

$$\begin{array}{cc} {\mathrm{Conv}}_{1\times 1}^{\mathrm{reduce}} & \to \mathrm{Norm}+\mathrm{SiLU}\to \mathrm{BasicStage}\;\left(\mathrm{LFE}\right)\\ & \to {\mathrm{Conv}}_{1\times 1}^{\mathrm{proj}}\to \mathrm{Gate}. \end{array}$$

#### 3.2.1. Local Feature Enhancement

**Figure 3:**
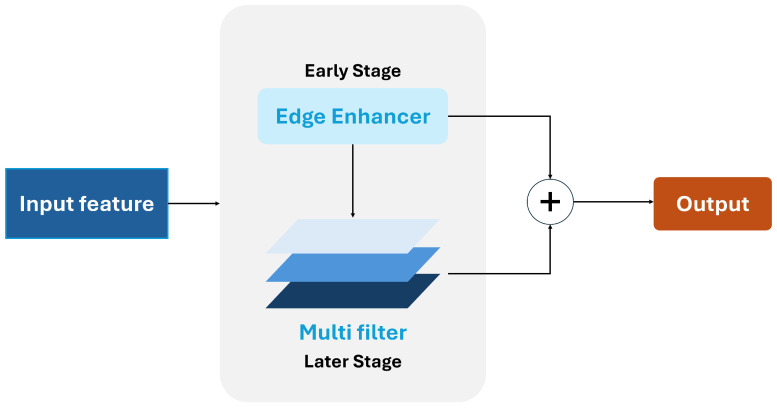
Stage-aware design of the Local Feature Enhancement (LFE) block, illustrating edge enhancement and noise suppression across different stages.

The Local Feature Enhancement (LFE) block is illustrated in Figure 3.

It is designed to explicitly model edge-sensitive structures that tend to be weakened in deep convolutional backbones. Instead of relying on a single fixed operator, LFE adopts a stage-aware design across multiple enhancement blocks, where each block is assigned a distinct role according to its relative depth within the module.

The early and later stages shown in Figure 3 correspond to the shallow and deeper LFE blocks within the BasicStage in Figure 2. Specifically, Stage 0 operates as the early stage for edge enhancement, while Stages 1–3 progressively transition to deeper stages focusing on noise suppression. In early stages, where features mainly encode low-level spatial details, LFE applies an Edge-Enhance module to strengthen fine structural patterns and boundary responses. This operation emphasizes high-frequency components that are crucial for detecting subtle defects but are often suppressed by standard convolutional downsampling. In later stages, as feature maps become more semantic and noise accumulated from previous layers may be amplified, LFE switches to a Noise Suppression Module. Specifically, the Noise Suppression Module is implemented using a depthwise Gaussian filtering operator with a fixed kernel, which performs spatial smoothing to attenuate high-frequency noise while preserving structural consistency. This module attenuates redundant high-frequency responses while preserving discriminative structural cues, producing cleaner representations for subsequent detection heads.

By adaptively balancing edge amplification and noise suppression across stages, the proposed LFE block captures complementary structural information while maintaining robustness to noise, enabling more reliable defect localization.

#### 3.2.2. Gated Module

Although the LFE blocks explicitly enhance edge-related responses, directly injecting enhanced features into the backbone may amplify noisy or irrelevant patterns, especially in texture-rich industrial images. To achieve stable integration, we introduce a gated module unit that uses edge-enhanced cues to recalibrate the original backbone activations in an in-place multiplicative manner, without introducing new feature patterns into the main stream.

Let $f_{0}\in R^{C\times H\times W}$ denote the original intermediate backbone feature at the C3 stage, and let *x* denote the output of the enhancement branch after ${\mathrm{Conv}}_{1\times 1}^{\mathrm{proj}}$. We predict a spatial importance map as follows:

(4) $$g=\sigma ({\mathrm{Conv}}_{1\times 1}\left(x\right)),$$

where $g\in {\left[0,1\right]}^{H\times W}$ indicates the relative importance of each spatial location and σ(·) is the sigmoid function. In practice, *g* serves as a spatial gain map that determines where the backbone responses should be strengthened. Based on this map, the final output is obtained through a multiplicative residual scaling:

(5) $$\mathrm{EdgeEnhance}\left(f_{0}\right)=f_{0}⊙\left(1+\alpha g\right),$$

where ⊙ denotes element-wise multiplication and α is a scaling factor (broadcast along the channel dimension). This formulation has two desirable properties. First, when g→0, the module degenerates to an identity mapping, i.e., $\mathrm{EdgeEnhance}\left(f_{0}\right)\approx f_{0}$, which preserves semantic consistency of the backbone and improves optimization stability. Second, when *g* takes large values around edge-dominant regions, the corresponding backbone activations are selectively amplified, yielding more compact and contour-aligned responses while suppressing unreliable activations caused by background textures or residual high-frequency noise. Equation (5) defines the mathematical formulation, while Algorithm 1 provides a step-by-step implementation of the proposed gated module.
**Algorithm 1** Implementation of gated module with in-place multiplicative recalibration**Require:** Backbone feature $f_{0}\in R^{C\times H\times W}$, enhanced feature *x*, scaling factor α**Ensure:** Recalibrated feature $\tilde{f}\in R^{C\times H\times W}$ 1: $g\leftarrow \sigma ({\mathrm{Conv}}_{1\times 1}\left(x\right))$▹$g\in {\left[0,1\right]}^{1\times H\times W}$ 2: $\tilde{f}\leftarrow f_{0}⊙\left(1+\alpha \cdot g\right)$▹ broadcast along channel dimension 3: **return** 
$\tilde{f}$

As a result, the proposed gated module improves robustness and training stability, leading to more controlled feature integration across different defect types.

### 3.3. Training Objective

YOLO-style detectors and RT-DETR are trained using the detection loss implemented in the Ultralytics framework, while Faster R-CNN follows its corresponding detection loss. For fair comparison, all reproduced models are trained and evaluated under the same data partition, input size, training schedule, augmentation setting, and evaluation protocol within each dataset.

## 4. Experiments

### 4.1. Experimental Environment and Evaluation Criteria

#### 4.1.1. Experimental Settings

All experiments are conducted on a workstation equipped with an NVIDIA RTX 4090 GPU. YOLO-style detectors and RT-DETR are implemented using the Ultralytics framework, while Faster R-CNN is reproduced using its corresponding detection pipeline. Two public steel surface defect datasets, NEU-DET and GC10-DET, are used for evaluation. NEU-DET is adopted as the primary benchmark for controlled comparison and ablation analysis, while GC10-DET is used as a supplementary evaluation under a different defect distribution. For the reproduced comparisons, Ultralytics-based detectors are initialized with official pretrained weights provided by the Ultralytics framework when available, while Faster R-CNN follows its corresponding official pretrained-weight setting. Within each dataset, all reproduced models are trained and evaluated under the same data partition, input size, training schedule, augmentation setting, and evaluation protocol to ensure controlled comparison.

#### 4.1.2. Dataset Description

To evaluate the proposed method on steel surface defect detection, two public benchmark datasets, NEU-DET and GC10-DET, are employed in our experiments.

##### NEU-DET Dataset

The NEU-DET dataset is collected by Northeastern University and is widely used in industrial surface defect detection research. It contains six typical defect categories, namely Crazing, Inclusion, Patches, Pitted Surface, Rolled-in Scale, and Scratches. The dataset consists of 1800 grayscale images with a resolution of 200×200 pixels, where each category includes 300 samples. Due to its relatively balanced class distribution and clean annotations, NEU-DET is suitable for fair performance comparison and ablation analysis. In this work, NEU-DET is adopted as the primary benchmark.

A class-balanced 3:1:1 hold-out protocol is adopted for NEU-DET. For each defect category, approximately 180 images are used for training, 60 images for validation, and 60 images for testing. The same split is fixed for all reproduced models, and all models are trained and evaluated under the identical data partition to ensure a fair comparison.

The detailed training settings for NEU-DET are listed in Table 1.

##### GC10-DET Dataset

The GC10-DET dataset contains ten steel surface defect categories with diverse visual patterns and more complex background textures than NEU-DET, including Punching Hole, Welding Line, Crescent Gap, Water Spot, Oil Spot, Silk Spot, Inclusion, Rolled Pit, Crease, and Waist Folding. Compared with NEU-DET, GC10-DET exhibits stronger class imbalance and higher intra-class variation, making it more challenging for defect detection. In this work, GC10-DET is used as supplementary cross-dataset evidence rather than as a claim of state-of-the-art generalization.

All reproduced models are trained and evaluated under the same fixed GC10-DET data partition and training protocol, with the detailed settings listed in Table 2. The input size is set to 640×640 to better preserve small defect patterns.

#### 4.1.3. Evaluation Metrics

We evaluate detection performance using Precision (*P*), Recall (*R*), and mean Average Precision (mAP). Specifically, AP summarizes the area under the precision–recall curve for each class, and mAP is obtained by averaging AP over all classes. We report mAP@0.5 as the primary metric, where a prediction is considered correct if its IoU with the ground-truth box exceeds 0.5. To describe model complexity, Params and FLOPs are reported. Inference latency and FPS are also reported for reproduced models under the same hardware environment. All metrics are computed on the fixed evaluation split with identical input resolution and evaluation settings for fair comparison.

### 4.2. Experimental Analysis

In this section, we report quantitative comparisons, runtime evaluation, ablation studies, and qualitative behaviors of EDEN-YOLO on the NEU-DET benchmark.

Table 3 presents a controlled comparison with reproduced detectors on NEU-DET, including a classical two-stage detector, an anchor-based one-stage detector, a DETR-style detector, and YOLO-style detectors. All models are trained and evaluated under the same data partition, input resolution, optimizer setting, augmentation strategy, and evaluation protocol to ensure a fair comparison.

All experiments were done under a unified training protocol. As shown in Table 3, EDEN-YOLO obtains higher mAP@0.5 than the reproduced YOLOv8 baseline under the same experimental protocol. Compared with the classical two-stage detector Faster R-CNN, EDEN-YOLO improves mAP@0.5 by 9.4 percentage points while using fewer parameters and FLOPs. Compared with RT-DETR, EDEN-YOLO improves mAP@0.5 by 9.2 percentage points and requires lower computational cost.

Among YOLO-style detectors, EDEN-YOLO improves the YOLOv8 baseline from 75.0% to 80.5% mAP@0.5, corresponding to an absolute gain of 5.5 percentage points. It also outperforms YOLOv12 by 4.6 percentage points, though EDEN introduces a moderate increase in computational cost compared with the two YOLO-style models mentioned above. These results indicate that the proposed Edge-Enhance module can improve edge-sensitive feature representation within a YOLO-style detector with an explicitly quantified increase in computational cost.

#### 4.2.1. PR Curve Analysis

**Figure 4:**
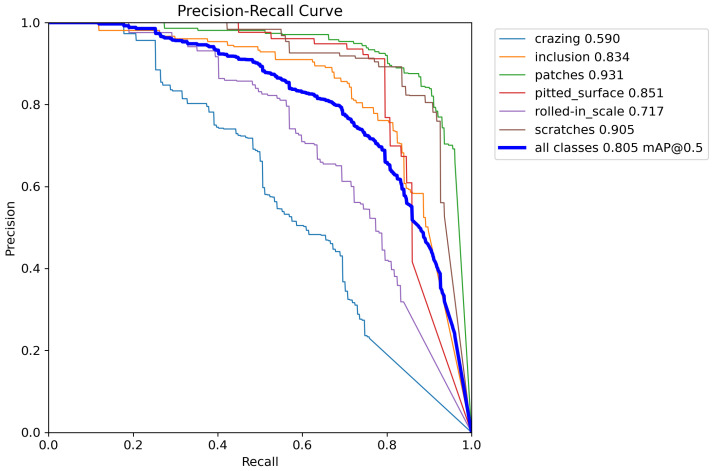
Precision–recall curves on NEU-DET.

Figure 4 further visualizes the precision–recall curves on NEU-DET. Overall, EDEN-YOLO maintains higher precision across a wide recall range, indicating improved localization confidence and reduced false positives compared with the baseline. These results validate the effectiveness of edge-aware recalibration at intermediate features for steel defect detection.

#### 4.2.2. Runtime Evaluation of Reproduced Models

Runtime comparison is produced among Faster-RCNN, RT-DETR, YOLOv8, YOLOv12, and EDEN-YOLO under the same inference setting.

Pre., Inf., Post., and Latency denote preprocessing time, inference time, postprocessing time, and total latency in milliseconds per image, respectively. FPS is calculated as 1000/Latency. All models are evaluated on the same hardware platform. For YOLOv8, YOLOv12, RT-DETR, and EDEN-YOLO, the same input size, batch size, and Ultralytics inference environment are used. Faster R-CNN follows its reproduced inference pipeline and input resolution, and its runtime is reported as an auxiliary reference because its proposal-based detection process differs from YOLO-style detectors.

As shown in Table 4, EDEN-YOLO achieves 68.0 FPS with a total latency of 14.7 ms/image under the tested environment. Compared with the YOLOv8 baseline, EDEN-YOLO introduces additional runtime cost due to the Edge-Enhance module. Nevertheless, its latency remains lower than those of RT-DETR and Faster R-CNN under the reproduced inference setting. These results indicate that EDEN-YOLO improves detection accuracy with an explicitly quantified additional inference cost, providing a clearer accuracy–efficiency trade-off rather than relying only on Params and FLOPs.

#### 4.2.3. Model Complexity Analysis

Compared with the YOLOv8 baseline, the parameter count increases from 3.01 M to 3.53 M, while the FLOPs increase from 8.2 G to 9.5 G. Although EDEN-YOLO introduces additional parameters due to the Edge-Enhance branch, the increase in model complexity is modest relative to the performance gain. Given the improvement in detection accuracy, this trade-off demonstrates that the proposed module enhances feature representation efficiency rather than relying on complex modules. In particular, the additional computation is concentrated at intermediate feature levels, where edge-aware recalibration provides the most benefit for detecting edge-dominant defects.

Together with the runtime results in Table 4, these results show that EDEN-YOLO improves detection accuracy over the reproduced YOLOv8 baseline while introducing additional but explicitly quantified computational cost.

### 4.3. Ablation Study

In this section, we investigate the impact of key design choices on model performance, including the insertion position of the Edge-Enhance module, component-level variants, fusion mechanisms, and the scaling factor α. Our goal is to evaluate the trade-off between performance gain and model complexity (in terms of both parameters and computational cost).

#### 4.3.1. Effect of Module Placement

To determine the optimal insertion position of the proposed Edge-Enhance module, we evaluate four single-stage insertion settings within the YOLOv8-style backbone. Specifically, C1, C2, C3, and C4 correspond to progressively deeper backbone features. In this ablation, the Edge-Enhance module is inserted into only one stage at a time, while all other training settings are kept unchanged. Multi-stage insertion is not considered because EDEN-YOLO aims to introduce a compact single-stage enhancement mechanism rather than stacking multiple enhancement branches.

Table 5 presents the quantitative results in terms of class-wise AP@0.5 and overall mAP@0.5.

As shown in Table 5, inserting the Edge-Enhance module at C3 achieves the best overall performance. Although the C2 setting obtains the highest AP@0.5 on Crazing, its performance on Rolled-in Scale decreases noticeably, resulting in a lower overall mAP@0.5 of 78.5%. This indicates that shallower features can preserve more fine-grained structural details, but they may also introduce stronger background interference and less stable category balance.

Compared with C1 and C2, the C3 feature provides a better trade-off between spatial resolution and semantic abstraction. It maintains sufficient structural information for edge-dominant defects while providing stronger semantic discrimination than shallower features. In contrast, the C4 setting relies on deeper features, where fine edge details may already be weakened by repeated downsampling. Therefore, C3 is selected as the final insertion position of EDEN-YOLO.

Overall, these results demonstrate that selective enhancement at the intermediate C3 stage achieves the most favorable balance between overall detection accuracy and class-wise stability.

#### 4.3.2. Component-Level Ablation of the Edge-Enhance Module

To further explore the contributions of individual components in the proposed Edge-Enhance module, we conduct component-level ablation experiments on the NEU-DET evaluation split. All variants are inserted at the C3 stage and trained under the same experimental protocol. The plain branch variant replaces the proposed stage-aware LFE branch with a standard convolutional branch, which is used to examine whether the improvement is merely caused by adding an extra branch. The w/o Gaussian and w/o Scharr variants remove the Gaussian noise-suppression operator and the Scharr edge operator from the full Edge-Enhance module, respectively. It should be noted that “w/o” denotes removing the corresponding component from the full Edge-Enhance module.

As shown in Table 6, the plain branch variant improves the YOLOv8 baseline from 75.0% to 78.4% mAP@0.5, indicating that introducing an intermediate recalibration branch is beneficial. However, the full EDEN-YOLO further improves the performance to 80.5%, demonstrating that the improvement is not merely caused by adding a standard convolutional branch. This result supports the effectiveness of the proposed stage-aware LFE design.

The w/o Gaussian variant achieves 79.2% mAP@0.5, which is lower than the full model. This indicates that the Gaussian noise-suppression operator contributes to stabilizing the edge-enhanced representation by reducing redundant high-frequency responses. Similarly, removing the Scharr operator decreases the overall mAP@0.5 to 78.7%, suggesting that explicit edge-sensitive gradient cues are important for defect localization. Although some ablated variants perform better on individual categories, the full EDEN-YOLO achieves the best overall mAP@0.5, confirming the complementary contribution of the stage-aware LFE branch, Scharr edge operator, Gaussian noise suppression, and gated recalibration.

#### 4.3.3. Effect of Fusion Mechanism

To evaluate the effectiveness of the proposed gated fusion mechanism, we compare it with a residual fusion strategy while keeping the local feature enhancement branch and the insertion position unchanged. The residual fusion variant directly combines the enhanced feature with the backbone feature, whereas the proposed gated fusion predicts a spatial importance map to selectively recalibrate the backbone response.

As shown in Table 7, the proposed gated fusion mechanism achieves better performance than the residual fusion strategy. Specifically, gated fusion improves mAP@0.5 from 76.0% to 80.5%, corresponding to a gain of 4.5 percentage points. Meanwhile, the gated fusion variant uses fewer parameters and lower FLOPs than residual fusion. This indicates that spatially selective recalibration is more effective than direct residual fusion for integrating edge-enhanced responses.

Compared with residual fusion, gated fusion does not directly inject all enhanced responses into the main feature flow. Instead, it uses a spatial importance map to determine where the original backbone feature should be strengthened. This selective mechanism helps suppress unreliable high-frequency background responses while preserving informative edge cues. Moreover, the gated fusion variant achieves higher accuracy with fewer parameters and lower FLOPs, indicating that the improvement does not come from increased model complexity but from a more effective feature fusion mechanism.

#### 4.3.4. Sensitivity Analysis of the Scaling Factor α

To investigate the influence of the scaling factor α in the Gated Module, we conduct a sensitivity analysis on the NEU-DET evaluation split. All variants are trained and evaluated under the same experimental configuration, and only the value of α is changed. The scaling factor controls the strength of gated recalibration in Equation (5), where a larger α leads to stronger amplification of selected spatial responses.

As shown in Table 8, the detection performance varies with the scaling factor α. When α is small, the gated recalibration is relatively weak, and the improvement brought by edge-sensitive enhancement is limited. As α increases to 0.7, EDEN-YOLO achieves the best overall performance, reaching 80.5% mAP@0.5. This setting also obtains the highest AP@0.5 on Crazing and Rolled-in Scale, which are closely related to edge-dominant and boundary-sensitive defect patterns.

When α is further increased to 0.9 or 1.0, the performance slightly decreases. This suggests that excessive feature amplification may introduce redundant high-frequency responses or background interference, thereby weakening the benefit of the Edge-Enhance module. Therefore, α=0.7 is adopted as the default setting in EDEN-YOLO. These results demonstrate that the scaling factor has a measurable influence on the Gated Module and that a moderate enhancement strength provides the best balance between structural enhancement and detection stability.

### 4.4. Visualization Analysis

**Figure 5:**
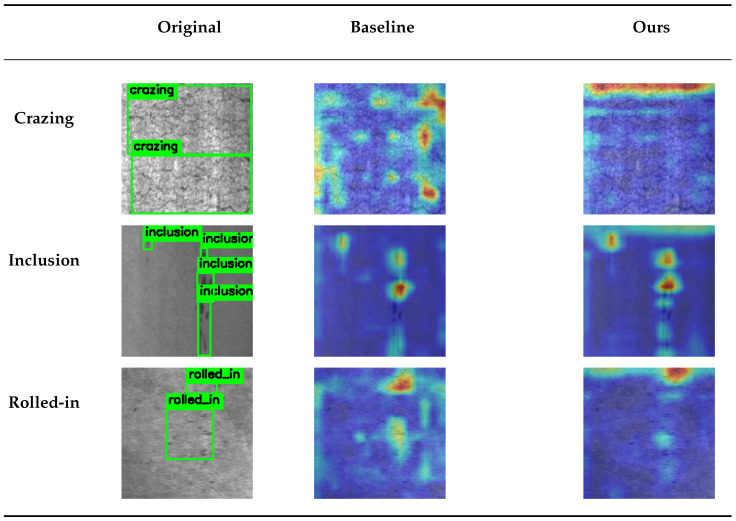
Qualitative comparison of feature responses (Part I). Rows: Crazing, Inclusion, Rolled-in. Columns: original image, baseline heatmap, and EDEN-YOLO heatmap. Warmer colors indicate stronger feature responses, whereas cooler colors indicate weaker feature responses.

**Figure 6:**
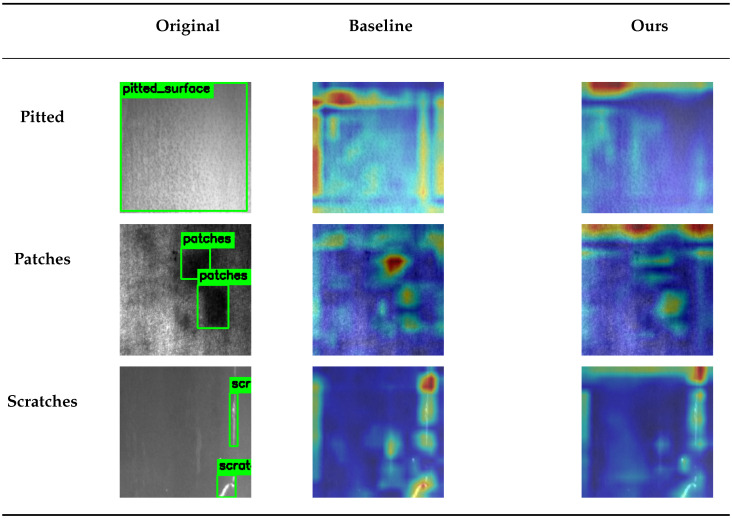
Qualitative comparison of feature responses (Part II). Rows: Pitted, Patches, Scratches. Columns: original image, baseline heatmap, and EDEN-YOLO heatmap. Warmer colors indicate stronger feature responses, whereas cooler colors indicate weaker feature responses.

Figure 5 and Figure 6 present qualitative comparisons of feature activation maps generated by the baseline and EDEN-YOLO. Overall, the baseline responses are often fragmented or biased toward textured background regions, whereas our model produces more compact and edge-aligned activations around defect contours, indicating enhanced structural sensitivity.

As shown in Figure 5, EDEN-YOLO exhibits particularly strong enhancement effects on defects with clear directional and edge-dominant characteristics, such as Crazing, Inclusion, and Rolled-in. For these categories, the proposed method generates continuous and intensified responses along thin and elongated edges, resulting in more complete contour coverage and reduced missing activations. In contrast, the baseline heatmaps tend to present scattered peaks or incomplete edge responses, which may lead to unstable localization under weak contrast conditions. These observations demonstrate that the proposed edge-aware enhancement effectively strengthens directional edge cues.

Figure 6 illustrates categories where the enhancement effect is relatively moderate, including Pitted Surface, Patches, and Scratches. For these texture-dominant or region-oriented defects, both the baseline and EDEN-YOLO produce relatively concentrated activations within defective areas. Nevertheless, our method still shows improved spatial consistency and reduced background interference, indicating that the enhancement branch does not simply amplify high-frequency noise.

We attribute these qualitative improvements to the in-place Edge-Enhance branch inserted at the intermediate C3 stage. The Local Feature Enhancement module extracts edge-aware structural cues, while the gated module adaptively controls the enhancement strength, suppressing unreliable responses in non-informative regions. Consequently, EDEN-YOLO achieves more discriminative and robust edge representations, providing visual evidence that complements the quantitative gains reported in previous sections.

### 4.5. Supplementary Evaluation on GC10-DET

The proposed model is further evaluated on GC10-DET. Compared with NEU-DET, GC10-DET contains more diverse defect patterns and exhibits stronger class imbalance and higher intra-class variation. In this paper, it is adopted as a supplementary experiment to test whether the model can maintain its detection ability under different defect backgrounds.

**Figure 7:**
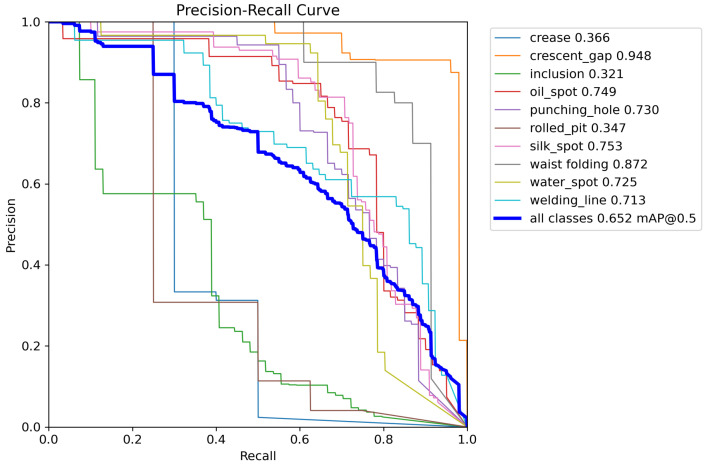
Precision–recall curves of EDEN-YOLO on the GC10-DET test split.

As shown in Table 9 and Figure 7, EDEN-YOLO achieves 65.2% mAP@0.5 on GC10-DET, improving over the reproduced YOLOv8 baseline by 4.2 percentage points. The gain is smaller than that on NEU-DET and is more class-dependent, which is consistent with the more complex defect distribution of GC10-DET. EDEN-YOLO performs favorably on Crescent Gap, Oil Spot, Silk Spot, Rolled Pit, and Waist Folding, while weak-contrast categories such as Inclusion and Crease remain challenging. This class-dependent behavior may be related to the imbalanced distribution of GC10-DET and the fact that the proposed module mainly enhances edge-sensitive structural cues.

Although the results show that EDEN-YOLO provides an overall improvement over the reproduced YOLOv8 baseline under a different defect distribution, the class-wise results also indicate that the benefit is not uniform across all defect types. This suggests that the proposed edge-aware recalibration is more beneficial for structure-sensitive defects, while weak-contrast or minority categories still require further investigation.

## 5. Conclusions

This paper presented EDEN-YOLO, a YOLO-based detector designed to enhance edge-sensitive representations for steel surface defect detection. Unlike approaches that rely on parallel branches or heavy attention modules, EDEN-YOLO introduces an in-place Edge-Enhance module at the C3 stage, where edge-related structural information can be utilized while maintaining a balance between spatial resolution and semantic abstraction. The module integrates a stage-aware Local Feature Enhancement (LFE) mechanism for capturing edge-sensitive structures and suppressing texture noise, together with a Gated Module(optimal α=0.7) for spatially selective multiplicative recalibration. This design enhances structural awareness while preserving the original YOLO-style backbone–neck–head topology.

Experiments on NEU-DET demonstrate consistent improvements over the reproduced YOLOv8 baseline under controlled experimental settings. EDEN-YOLO achieves 80.5% mAP@0.5 on the NEU-DET evaluation split while introducing additional inference cost compared with YOLOv8, as explicitly reported by the runtime evaluation. Supplementary evaluation on GC10-DET further shows that EDEN-YOLO obtains 65.2% mAP@0.5 and improves over the reproduced YOLOv8 baseline under the same GC10-DET protocol, although the improvement is more class-dependent than on NEU-DET. Qualitative visualizations further show that the proposed method produces more compact and edge-aligned responses, indicating improved suppression of background interference.

Despite these advantages, several limitations should be noted. EDEN-YOLO is not claimed to achieve absolute state-of-the-art performance over all literature-reported detectors. Second, the Edge-Enhance module introduces additional inference complexity compared with the YOLOv8 baseline. Third, the GC10-DET results are used as supplementary evaluation under a different defect distribution, and the class-wise results indicate that weak-contrast and minority categories such as Inclusion and Crease remain challenging. Broader evaluation on larger and more diverse industrial defect datasets will be an important direction for future work.

## Figures and Tables

**Table 1:** Experimental parameter settings.

Parameter	Setup
Epochs	220
Input size	320×320
Batch size	8
Initial learning rate	$5\times 10^{-4}$
Final learning rate	$5\times 10^{-6}$
Optimizer	AdamW
Weight decay	$5\times 10^{-4}$

**Table 2:** Experimental parameter settings for GC10-DET.

Parameter	Setup
Epochs	200
Input size	640×640
Batch size	8
Initial learning rate	$1\times 10^{-2}$
Final learning rate	$5\times 10^{-4}$
Optimizer	SGD
Weight decay	$5\times 10^{-4}$

**Table 3:** Controlled comparison of reproduced models on the NEU-DET dataset under the same experimental protocol.

Method	Cr	In	Pa	Pi	Ro	Sc	mAP@0.5	Params (M)	FLOPs (G)
Faster R-CNN	37.4	79.4	85.3	81.5	54.5	88.2	71.1	28.33	250.2
RT-DETR	37.7	70.9	85.1	80.5	64.1	89.7	71.3	42.77	130.5
YOLOv8	38.7	82.8	**94.0**	76.2	67.6	90.8	75.0	3.01	8.2
YOLOv12	43.4	80.3	93.9	81.8	61.6	**94.4**	75.9	2.57	6.5
EDEN-YOLO	**59.0**	**83.4**	93.1	**85.1**	**71.7**	90.5	**80.5**	3.53	9.5

Cr, In, Pa, Pi, Ro, and Sc denote Crazing, Inclusion, Patches, Pitted Surface, Rolled-in Scale, and Scratches, respectively. Bold indicates the best result in each column.

**Table 4:** Runtime comparison of reproduced models under the same inference setting.

Method	Pre. (ms)	Inf. (ms)	Post. (ms)	Latency (ms)	FPS
Faster R-CNN	5.2	17.8	1.5	24.5	40.9
RT-DETR	0.7	19.8	1.2	21.7	46.1
YOLOv8	0.9	3.5	2.1	6.5	153.8
YOLOv12	0.9	9.4	2.5	12.8	78.1
EDEN-YOLO	1.1	11.4	2.2	14.7	68.0

**Table 5:** Ablation study on the insertion position of the Edge-Enhance module on the NEU-DET evaluation split.

Setting	Crazing	Inclusion	Patches	Pitted Surface	Rolled-in Scale	Scratches	mAP@0.5
C1	55.1	79.3	90.4	82.5	64.3	93.4	77.5
C2	**59.1**	82.8	92.1	83.4	60.0	**93.5**	78.5
C3	59.0	**83.4**	**93.1**	**85.1**	**71.7**	90.5	**80.5**
C4	55.0	82.9	92.1	83.8	64.4	88.7	77.8

Bold indicates the best result in each column.

**Table 6:** Component-level ablation study of the Edge-Enhance module on the NEU-DET evaluation split.

Variant	Cr	In	Pa	Pi	Ro	Sc	mAP@0.5
YOLOv8 baseline	38.7	82.8	**94.0**	76.2	67.6	90.8	75.0
Plain branch	54.8	82.6	92.2	**85.5**	65.4	89.8	78.4
w/o Gaussian	57.1	83.0	92.2	82.1	68.6	**92.3**	79.2
w/o Scharr	**60.0**	82.5	92.6	76.8	68.7	91.6	78.7
Full EDEN-YOLO	59.0	**83.4**	93.1	85.1	**71.7**	90.5	**80.5**

Cr, In, Pa, Pi, Ro, and Sc denote Crazing, Inclusion, Patches, Pitted Surface, Rolled-in Scale, and Scratches, respectively. Bold indicates the best result in each column.

**Table 7:** Ablation study on different feature fusion mechanisms in EDEN-YOLO on the NEU-DET evaluation split.

Fusion Mechanism	Gate	mAP@0.5	Params (M)	FLOPs (G)
Residual Fusion	**✗**	76.0	3.78	10.2
Gated Fusion	**✓**	80.5	3.53	9.5
Δ	–	+4.5	−0.25	−0.7

**✓** indicates that the gate mechanism is used, whereas **✗** indicates that the gate mechanism is not used.

**Table 8:** Sensitivity analysis of the scaling factor α on the NEU-DET evaluation split.

α	Cr	In	Pa	Pi	Ro	Sc	mAP@0.5
0.1	49.9	82.9	**94.0**	82.3	64.5	92.5	77.7
0.3	55.6	82.5	92.5	81.0	68.1	90.5	78.4
0.5	55.2	82.4	93.0	77.6	63.2	**94.4**	77.6
0.7	**59.0**	**83.4**	93.1	**85.1**	**71.7**	90.5	**80.5**
0.9	55.3	82.8	93.4	83.8	66.1	92.7	79.0
1.0	55.0	83.3	93.4	**85.1**	64.5	90.7	78.7

Cr, In, Pa, Pi, Ro, and Sc denote Crazing, Inclusion, Patches, Pitted Surface, Rolled-in Scale, and Scratches, respectively. Bold indicates the best result(s) in each column.

**Table 9:** Controlled comparison of reproduced models on the GC10-DET dataset under the same experimental protocol.

Method	Pu	Wl	Cg	Ws	Os	Ss	In	Rp	Cr	Wf	mAP@0.5
Faster R-CNN	**86.0**	**97.4**	86.1	55.2	65.3	68.9	16.9	10.5	52.7	81.8	62.1
RT-DETR	67.1	51.9	78.0	80.3	64.5	50.7	29.9	9.5	**59.0**	73.9	56.5
YOLOv12	70.1	65.9	84.9	**85.1**	65.0	68.8	**43.9**	32.5	49.9	79.0	64.5
YOLOv8	75.0	59.5	86.5	83.4	63.2	68.7	36.9	24.7	33.7	78.8	61.0
EDEN-YOLO	73.0	71.3	**94.8**	72.5	**74.9**	**75.3**	32.1	**34.7**	36.6	**87.2**	**65.2**

Pu, Wl, Cg, Ws, Os, Ss, In, Rp, Cr, and Wf denote Punching Hole, Welding Line, Crescent Gap, Water Spot, Oil Spot, Silk Spot, Inclusion, Rolled Pit, Crease, and Waist Folding, respectively. Bold indicates the best result in each column.

## Data Availability

The original contributions presented in this study are included in the article. Further inquiries can be directed to the corresponding author.
